# EnDuSecFed: an ensemble approach for privacy preserving Federated Learning with dual-security framework for sustainable healthcare

**DOI:** 10.3389/fdata.2025.1659026

**Published:** 2026-01-22

**Authors:** Bela Shrimali, Jenil Gajjar, Swapnoneel Roy, Sanjay Patel, Kanu Patel, Ramesh Ram Naik

**Affiliations:** 1 Unitedworld Institute of Technology, Karnavati University, Gandhinagar, Gujarat, India; 2 Department of Computer Science and Engineering, Institute of Technology, Nirma University, Ahmedabad, Gujarat, India; 3 School of Computing, University of North Florida, Jacksonville, FL, United States

**Keywords:** Federated Learning, Fernet Symmetric Encryption, Intrusion Detection System, Logistic Regression, Random Forest, Support Vector Classifier

## Abstract

Recent advances in Artificial Intelligence have highlighted the role of Machine Learning in healthcare decision-making, but centralized data collection raises significant privacy risks. Federated Learning addresses this by enabling collaborative training across multiple clients without sharing raw data. However, Federated Learning remains vulnerable to security threats that can compromise model reliability. This paper proposes a dual-security Federated Learning framework that integrates Fernet Symmetric Encryption for secure transmission of model updates using symmetric encryption and an Intrusion Detection System to detect anomalous client behavior. Experiments on a publicly available healthcare dataset show that the proposed system enhances privacy and robustness compared to traditional FL. Among tested models, including Logistic Regression, Random Forest, and SVC, the ensemble method achieved the best performance with 99% accuracy.

## 1 Introduction

According to the Gartner report-2025 (Gartner, 2025), about 27% of organizations have faced a privacy breach or security issue related to Artificial Intelligence (AI). This means that there were intentional attacks on the organization's AI systems because they collect and process data in a central place. Federated Learning (FL) has emerged as a robust method for training machine learning models across multiple clients while maintaining the privacy of their local data. Unlike traditional methods where data is collected in one location, FL allows each client to have control of its data (Wang et al., 2023b). This is particularly useful in sensitive areas like healthcare, where patient information must be kept confidential (Khatun et al., 2023; Almalawi et al., 2023; Naresh and Thamarai, 2023). By using FL, healthcare providers can create better models by combining knowledge from different datasets without risking the privacy and security of individual patient data (Chaddad et al., 2023; Joshi et al., 2022; Kumar and Singla, 2021).

Despite its advantages, FL presents several challenges. Its decentralized architecture can introduce security vulnerabilities, particularly in securing the updates exchanged between clients and the central server. In a standard FL framework, the local model weights from each client are aggregated to form a global model. This aggregation process, however, is susceptible to security threats (Li et al., 2023; Coelho et al., 2023; Ali et al., 2024), such as data poisoning and adversarial attacks, which can compromise the performance of the global model. Such concerns are especially critical in healthcare applications, where prediction accuracy directly impacts patient safety.

To manage these risks, encrypted communication using the Fernet Symmetric Encryption (FSE) technique is implemented during the sharing of model updates between local clients and the global server. FSE allows secure calculations on encrypted data, ensuring that the model updates shared remain private. With FSE, the system protects sensitive information from attackers while still allowing clients to work together. This means even if a malicious client tries to change its model updates, the encryption will stop it from damaging the global model. While FSE secures model updates during sharing, it does not automatically detect malicious behavior or unusual activity in the Federated Learning system. Attackers can still send harmful updates that may compromise the global server. To address this, an Intrusion Detection System (IDS) is deployed at the global server to monitor and analyze incoming model updates for suspicious activity. By identifying abnormal patterns, the IDS can detect attacks such as model poisoning. This combined approach—using FSE for secure sharing and IDS for anomaly detection—enhances the overall security and trustworthiness of the FL process.

### 1.1 Motivation

Preserving the privacy of sensitive information is critical in healthcare, and FL has emerged as a promising paradigm as it enables collaborative model training without sharing raw data. Nevertheless, FL remains vulnerable to security threats, where malicious clients may submit harmful updates that compromise the global model's accuracy. This study aims to strengthen FL security in healthcare, where reliability is crucial for patient care. To address these challenges, Fernet Symmetric Encryption (FSE) is employed to safeguard model updates against tampering, while an IDS at the central server detects anomalous client behavior. The main contributions of this research are:

- We propose a federated learning method with dual security. A communication between local clients and the main server is secured using FSE and protects data changes at the central server with an IDS. Our method is shown to be better in security analysis compared to existing methods.
- To improve decision-making and predictions, along with existing models, an ensemble approach is also implemented that combines predictions from three main models: Logistic Regression, Support Vector Classifier, and Random Forest at the local node for training.
- We also discuss various attacks on privacy in FL models and highlight how our dual security approach adds value to this research area.

### 1.2 Organization

The remainder of this paper is structured as follows. Section 2 presents a comprehensive review of the existing literature. Section 3 details the proposed system architecture, including methodology, system components, and their interactions. Section 4 describes the experimental setup with the description of dataset, models and proposed algorithms. Section 5 provides an in-depth security analysis of the FSE scheme and IDS components, examining potential vulnerabilities and their mitigations. Section 6 presents experimental results, including performance metrics, comparative analysis, and validation of the approach. Lastly, Section 7 conclude with the key findings, discusses the implications of the work, and outlines promising directions for future research in this domain.

## 2 Literature review

FL has emerged as a promising privacy-preserving paradigm, particularly in sensitive domains such as healthcare. Unlike centralized machine learning, FL enables distributed model training without directly sharing raw data, thus safeguarding patient privacy. However, despite its advantages, FL remains vulnerable to adversarial threats, including data poisoning, label-flipping, and model poisoning attacks, where malicious clients can manipulate updates to reduce the performance of the global model (Hiwale et al., 2023). To address these vulnerabilities, researchers have explored various privacy-enhancing and security-aware strategies, which can be broadly categorized into: privacy-preserving approaches, cryptographic frameworks, IDS integration, and blockchain-enabled solutions. Privacy-preserving and cryptographic approaches.

Alazab et al. (2023) investigated FL for privacy-preserving Intrusion Detection Systems, comparing its performance against traditional deep learning models. By using the FedAvg algorithm, autoencoder-based anomaly detection, and secure gRPC channels, they reported high accuracy (98.07%), precision (97.4%), recall (99.06%), and F1-score (98.21%). Similarly, Wang et al. (2023a) introduced PPFLHE, a framework that leverages homomorphic encryption to address privacy and communication overhead in healthcare FL. Their system achieved 81.53% accuracy, showing that encryption can secure model updates but may also introduce computational overhead.

To mitigate adversarial threats, Almalki et al. (2024) proposed a hybrid Healthcare 5.0 framework that combines FL, IDS, and Blockchain Technology (BCT). Their solution improved diagnostic accuracy (93.89%) while enhancing data protection in Internet of Medical Things (IoMT) applications. Schneble (2018) explored FL-based distributed IDS for Medical Cyber-Physical Systems (MCPS), focusing on detecting cyberattacks while maintaining high accuracy and low false-positive rates. Guduri et al. (2023) further advanced security in FL by integrating blockchain with lightweight encryption and proxy re-encryption to secure Electronic Health Records (EHR). Their Ethereum-based testbed demonstrated superior resistance to unauthorized access compared with existing models.

While this literature demonstrates significant progress, several gaps remain. Privacy-preserving approaches like homomorphic encryption and FSE secure data during communication but do not inherently detect malicious updates, leaving models vulnerable to model poisoning. IDS-based solutions focus on anomaly detection but face challenges in scalability and false alarms in highly distributed healthcare environments. Blockchain-enhanced systems improve auditability and decentralization but often introduce high computational and communication overhead. Furthermore, many proposed frameworks are evaluated on limited datasets or focus primarily on accuracy, with less emphasis on robustness against adaptive adversaries or combined privacy–security trade-offs.

From this review, it is evident that while existing literature addresses either privacy (via encryption/FSE) or security (via IDS/blockchain), very few frameworks offer a comprehensive and lightweight defense mechanism that jointly ensures secure sharing of updates and real-time detection of adversarial behaviors in FL for healthcare applications. This gap motivates our research, where we propose an integrated approach combining FSE for privacy-preserving updates with a global IDS for anomaly detection, thereby enhancing the trustworthiness of FL in sensitive healthcare settings.

Table 1 provides a summary of the existing state-of-the-art in FL for healthcare applications, highlighting their contribution, limitations, technologies used, comparison parameters, and security concerns/attacks discussed.

**Table 1:** Review of existing research in privacy-preserving Federated Learning.

**Existing Work**	**Contribution**	**Limitations**	**Technology Used**	**Performance Metrics**	**Attacks Considered**
Abaoud et al. (2023)	Privacy-preserving FL models	Scalability issues	DP, FSE, HE	Acc.: 97.69%, Prec.: 95.2%, Rec.: 93%	None reported
Chen et al. (2023)	PPTFL model ensuring traceable and tamper-proof parameters	Computational complexity, overhead	BCT, IPFS, CNN, ResNet-18	CNN: 91.46%, ResNet-18: 68.76%	Backdoor attacks
Gayathri Hegde et al. (2023)	Comparison of various FL models	High processing time for ANN	ANN, LR	FL-LR: 98.12%, FL-ANN: 97.66%	None reported
Shen et al. (2023)	Privacy-preserving online diagnosis scheme for e-healthcare systems	Computational complexity, scalability issues	SVM, HE	Acc.-1: 86.4%, Acc.-2: 85.9%, Acc.-3: 90.7%	None reported
Otoum et al. (2021)	Federated Reinforcement Learning-based IDS for IoT in healthcare	Scalability challenges	RL, SVM	FRL-IDS: 98%, SVM: 98.5%	DoS, DDoS, Web Attacks (XSS, SQL Injection, Brute Force), HeartBleed, PortScan
Alazab et al. (2023)	Evaluated FL effectiveness in privacy-preserving IDS	Not specified	FedAvg, Autoencoder, gRPC	Acc.: 98.07%, Prec.: 97.4%, Rec.: 99.06%, F1: 98.21%	None reported
Wang et al. (2023a)	PPFLHE framework for healthcare data security	Communication overhead	Homomorphic Encryption	Acc.: 81.53%	Internal attacks, Chosen-Plaintext
Almalki et al. (2024)	Secure Healthcare 5.0 system integrating FL, IDS, and BCT	Not specified	FL, IDS, BCT	Acc.: 93.89%	None reported
Schneble (2018)	Distributed ML-based IDS for Medical CPS	Not specified	FL	High detection accuracy, low false positives (exact values not provided)	DoS, Data modification, Data injection
Guduri et al. (2023)	Blockchain-based FL for EHR security	Not specified	Lightweight encryption, Decentralized cloud, Proxy re-encryption	Improved security metrics (values not provided)	Message tampering, Replay, Man-in-the-middle
Srivenkateswaran et al. (2025)	ECC-Serpent hybrid encryption scheme	Integrating Serpent encryption may introduce additional computational overhead compared to lightweight algorithms.	ECC, Hybrid encryption model	97.5% accuracy in safeguarding sensitive healthcare data	Passive attack, Reply attack

## 3 Proposed architecture

This section covers the discussion on FL and proposed architecture along with security mechanisms, i.e, FSE and IDS, in separate subsections.

### 3.1 Overview of the architecture

**Figure 1:**
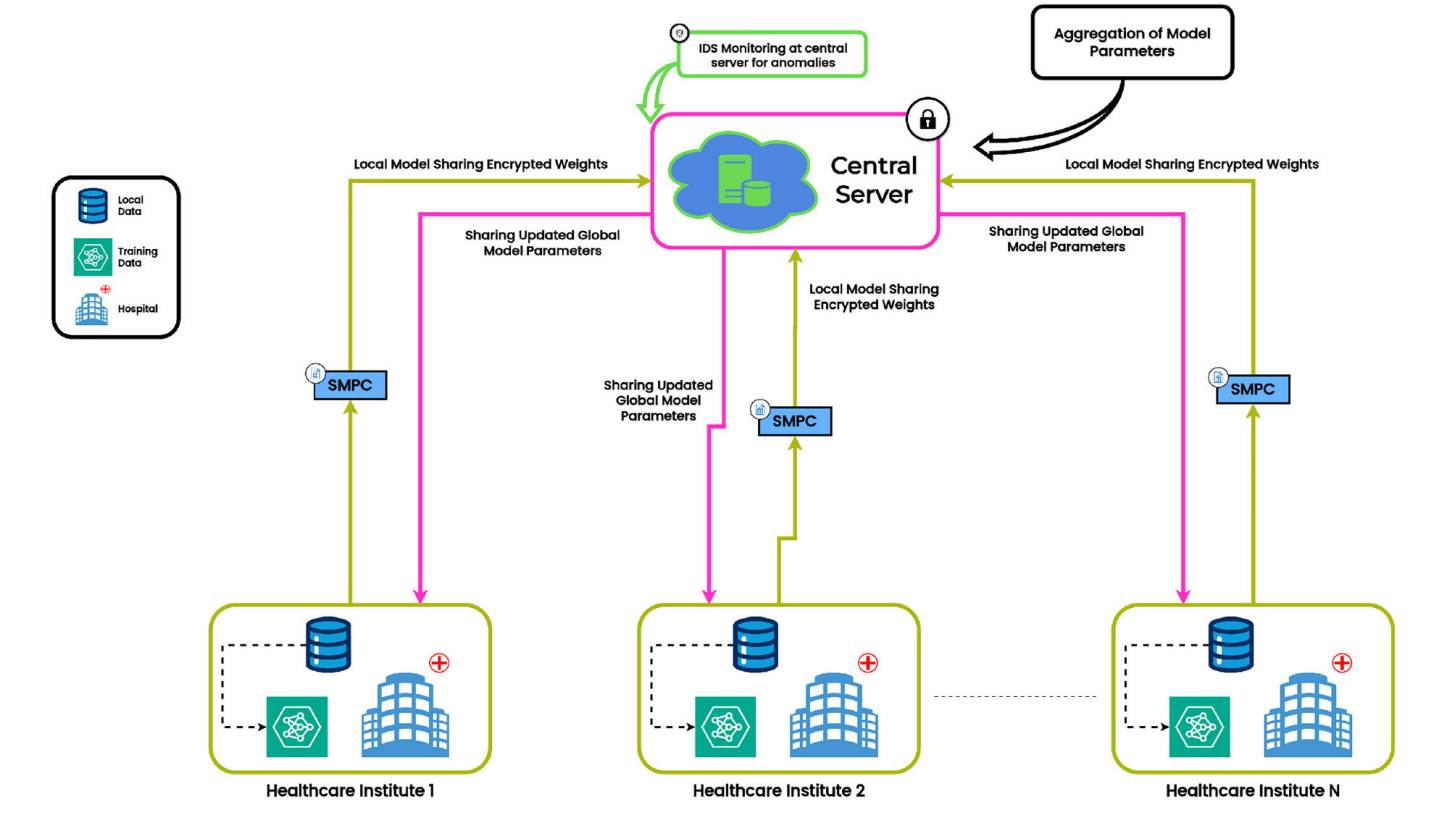
Federated Learning architecture. (i) Local Nodes train models on their on data. (ii) Secure Multi-Part Computation encrypts the model updates before transmitting them to the central server. (iii) Local Nodes send their encrypted model updates to the central server. (iv) The central server decrypts and aggregates the updates to update the global model. (v) The central server uses its IDS to monitor for any abnormalies in the decrypted updates, ensuring integrity against malicious contributions.

Figure 1 illustrates a Federated Learning (FL) framework used in healthcare facilities, having an IDS and FSE to guarantee security throughout the communication and learning process. The process/ steps of the proposed work, as shown in Figure 1 are as follows:

- **Local model training**: Each medical facility (e.g., Healthcare Institute 1, 2, 3,... N) uses the infrastructure of the organization to process its local dataset and train a machine learning model. This guarantees the confidentiality of the patient's information. The training procedure closely complies with privacy-protecting guidelines.
- **Local model sharing with FSE**: After training, updates to the local model are encrypted, then sent to the central server via FSE. By preventing unwanted access or tampering, this encryption guarantees that the model updates remain secure while in transit. Malicious local nodes trying to deduce private information during communication are another risk that the FSE reduces.
- **Global model aggregation**: To create a global model, the central server gathers the encrypted weights that are received from each participating local node and decrypts them. The central server accurately aggregates the contributions of local nodes without introducing any malicious activity because it is presumed to be non-malicious.
- **Global IDS monitoring**: An IDS at the central server is used to keep an eye out for irregularities in decrypted model updates, even though the server is reliable. To make sure they don't have a detrimental effect on the global model, the IDS detects and flags suspicious updates coming from potentially malicious local nodes, such as those with extreme model parameter deviations.
- **Global Model Distribution**: Following aggregation, each local healthcare facility receives a copy of the global model. To increase the precision of its forecasts, every institution makes use of the recent global model.

The proposed architecture ensures that malicious activity coming from local nodes is identified and stopped before it can compromise the integrity of the global model by combining FSE for secure communication with an IDS for anomaly detection.

### 3.2 Working of federated learning framework

A decentralized machine learning technique called federated learning allows several devices or organizations (Oh and Nadkarni, 2023) to work together to train a model without exchanging raw data. Multiple local nodes and a global node make up an FL's two ends, with the client servers keeping their local data and the central server maintaining the global model (Islam et al., 2023). Each client uses its data in the paradigm to train the model locally; only the central server receives the model weights for aggregation. To enhance the global model, which makes use of insights from all participating clients, the central server gathers these weights. Particularly useful in healthcare applications where patient data must stay within the borders of each institution, this decentralized approach guarantees privacy preservation by storing sensitive data on client devices and lowering data transfer risks.

The aggregated global model weights are calculated using Equation 1, which represents the federated averaging mechanism:


$$\begin{array}{l} W_{\text{global}}=\frac{1}{N}\overset{N}{\underset{i=1}{\sum }}W_{\text{local},i} \end{array}$$ (1)

where *W*_global_ is the global model weight, *W*_local_ represents the local weights of client *i*, and *N* is the total number of clients. This equation ensures that each client's contribution is equally weighted in the global model, providing a democratic aggregation approach where no single client dominates the learning process.

### 3.3 Secure transmission of model updates using symmetric encryption

FSE is a cryptographic technique that enables a node to authenticate and encrypt messages between parties (Sadu, 2024). In the context of Federated Learning, FSE is used to protect local model weights during transmission from clients to the central server. Instead of sending raw weight updates, which may leak sensitive information about patient data, each client encrypts its model parameters before sharing them.

Mathematically, the process can be described as follows. For a given client *i*, the local model weights *W*_local, *i*_ are encrypted before transmission:


$$\begin{array}{l} W_{\text{encrypted}}=\text{Encrypt}\left(W_{\text{local}},K_{\text{FSE}}\right) \end{array}$$ (2)

where *K*_FSE_ is the secret encryption key (or a set of keys, in the case of threshold cryptography). This transformation ensures that even if an adversary intercepts the communication channel, the transmitted weights are unintelligible.

At the server side, decryption is performed to recover the original updates:


$$\begin{array}{l} W_{\text{decrypted}}=\text{Decrypt}\left(W_{\text{encrypted}},K_{\text{FSE}}\right) \end{array}$$ (3)

This allows the central server to aggregate weights securely while ensuring that no raw data is ever exposed.

#### 3.3.1 Key properties and guarantees

The use of FSE in our framework provides several important guarantees:

- **Confidentiality:** Local model updates remain private during transmission, preventing leakage of patient-level data.
- **Collusion resistance:** Even if multiple clients collude, they cannot recover another client's raw data, as only encrypted updates are visible in transit.
- **Integrity of transmission:** By coupling encryption with authentication tags (e.g., Fernet symmetric encryption), tampering with updates can be detected.

#### 3.3.2 Implementation considerations

In our implementation, the FSE scheme was employed, which provides both confidentiality and authentication. Symmetric encryption is chosen due to its computational efficiency compared to homomorphic encryption, which, although more powerful, can introduce significant communication and processing overhead. The global server generates and securely distributes the shared encryption key *K*_FSE_ to each participating client during initialisation, ensuring that all parties can participate in secure encryption and decryption.

While FSE secures communication channels, it does not by itself detect malicious updates (e.g., model poisoning). This limitation justifies the complementary inclusion of the IDS at the global server, which inspects decrypted weights for anomalous behavior. Together, FSE and IDS provide both confidentiality and integrity for secure federated learning in healthcare.

### 3.4 Intrusion detection system

An IDS is a security tool that monitors and analyzes system activity to detect suspicious behavior, unauthorized access, or cyberattacks (Mosaiyebzadeh et al., 2023). Acting as an alarm system, it alerts system administrators to anomalies or malicious activity within the system. In the context of FL, the IDS safeguards the training process by detecting malicious or unusual behavior. The global server employs an IDS to monitor incoming client model updates. Using anomaly detection techniques, it identifies inconsistencies—such as significant deviations in model parameters—that may indicate malicious activity. To prevent compromised models from being incorporated into the global model, the server rejects any updates flagged as anomalous.

The anomaly detection technique used here checks for unusual changes in the model's weights as defined in Equation 4:


$$\begin{array}{l} \text{Anomaly Detected if }||{\text{w}}_{i}-{\text{w}}_{t}||>\delta \end{array}$$ (4)

In Equation 4, **w**_*i*_ represents the weight updates from client *i*, **w**_*t*_ is the current global model weights, and δ is the predefined threshold. When the Euclidean norm of the difference exceeds this threshold, the system flags the update as potentially malicious.

#### 3.4.1 Threshold selection

The threshold value δ plays a critical role in balancing sensitivity and false alarms. In our experiments, δ was set empirically based on the distribution of update magnitudes across clients, with values chosen around the 95th percentile of observed deviations during benign training. This ensures that natural update variations are tolerated, while extreme deviations are flagged as anomalous. In practical deployments, δ can be dynamically adapted using validation rounds or statistical confidence intervals, making the IDS adaptable to different datasets and model architectures.

#### 3.4.2 Need for IDS alongside FSE

Although FSE encrypts the data during transmission to guarantee the privacy and confidentiality of the model weights, it lacks a way to ensure the data's integrity. Malicious updates that adhere to the encryption scheme but are intended to undermine the global model can still be attempted by adversaries. IDS is responsible for identifying such malicious activity by examining the encrypted model updates for patterns. By examining system behavior and contrasting it with a baseline of typical activity, an anomaly-based IDS can detect possible threats (Schneble, 2018). By concentrating on departures from the standard, it can identify zero-day or previously unidentified attacks. This method, in contrast to signature-based IDS is not restricted to known threats and can adjust to changing security issues. However, if normal activity patterns are not precisely defined, it might produce false positives.

## 4 Implementation

### 4.1 Dataset overview

The Lung Cancer Risk Detection (Biswas and Nath, 2024) dataset is used for proposed work. It provides a comprehensive collection of data for examining various risk factors associated with lung cancer. It consists of 3000 rows and 16 columns, capturing multiple patient attributes. Key features include **GENDER, AGE, SMOKING, ANXIETY, SHORTNESS_OF_BREATH, YELLOW_FINGERS, ALLERGY, ALCOHOL_CONSUMING, COUGHING, CHEST_PAIN**. A summary of the dataset is presented in Table 2.

**Table 2:** Summary of the lung cancer risk detection dataset.

**Property**	**Description**
Dataset name	Lung cancer risk detection dataset (Biswas and Nath, 2024)
Number of instances	3,000 patient records
Number of features	16 attributes (demographic, behavioral, psychological, and clinical)
Feature types	Categorical (e.g., gender, smoking, alcohol consuming), Numerical (e.g., Age), Binary/symptom indicators (e.g., shortness_of_breath, chest_pain, yellow_fingers, coughing, allergy)
Target variable	Presence or absence of lung cancer
Unique characteristics	Includes lifestyle habits, clinical symptoms, and psychological attributes (e.g., anxiety)

### 4.2 Local model discription

In the FL environment, each participating client—such as hospitals or diagnostic facilities—trains a local model on its private dataset without disclosing sensitive patient information. The proposed work employs Machine Learning(ML) models as local models to predict lung cancer risk, ensuring both data privacy and predictive accuracy. ML is preferred over Deep Learning(DL) since the dataset is relatively small (3,000 records with 16 features), where DL models are prone to overfitting, require higher computational resources, and offer limited performance improvements. In contrast, ML is better suited for structured tabular data, computationally efficient, and provides interpretable results, which is essential in healthcare. Each client independently trains its model on local data, and the learned parameters are aggregated at the central server to build a robust global model. Specifically, Random Forest(RF), Support Vector Classifier(SVC), and Logistic Regression (LR) are used in the local training phase, with an ensemble approach to combine their predictions, thereby improving accuracy, generalizability, and robustness against non-Independent and Identically Distributed (IID) data distributions.

All ML models were trained with model-specific hyperparameters. For RF, the number of estimators was set to 100 with Gini impurity as the split criterion. For SVC, an RBF kernel was used with *C* = 1.0 and γ = scale. For LR, the solver was set to “liblinear” with L2 regularization and a maximum of 1,000 iterations. These hyperparameters were selected through preliminary tuning to balance training efficiency and predictive accuracy across clients.

The following subsections describe these classifiers in detail and their role in the federated setup.

#### 4.2.1 Random forest

**Figure 2:**
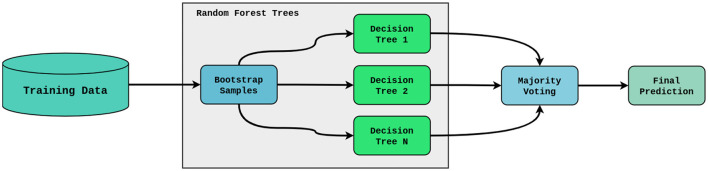
Random forest architecture.

An ensemble learning technique called Random Forest builds several decision trees during training and produces a class that is the average of the classes of the individual trees. The Random Forest model will use 16 features in the dataset to produce a strong predictive model for lung cancer risk detection as shown in Figure 2.

#### 4.2.2 Support vector classifier

**Figure 3:**
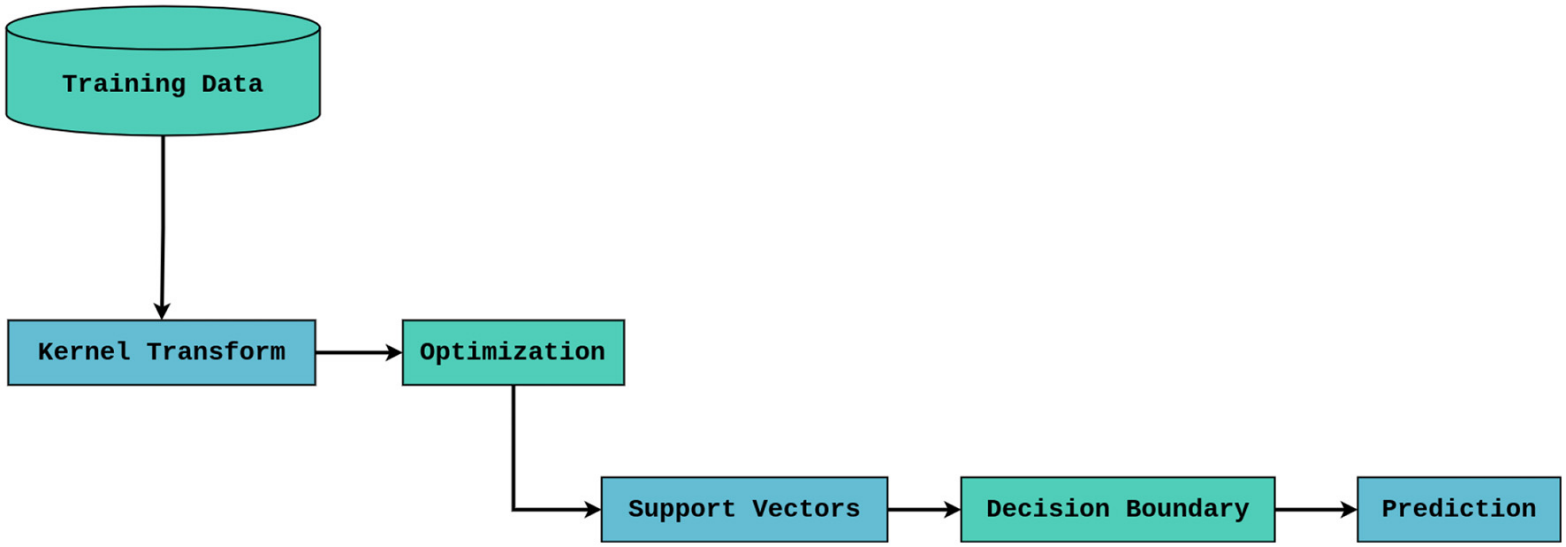
Support vector classifier architecture.

The goal of the Support Vector Classifier (SVC) is to identify the best hyperplane in the feature space for dividing the various classes. The SVC will attempt to maximize the margin between the classes by mapping the 16 input features into a high-dimensional space in the context of lung cancer risk detection as shown in Figure 3.

#### 4.2.3 Logistic regression

**Figure 4:**
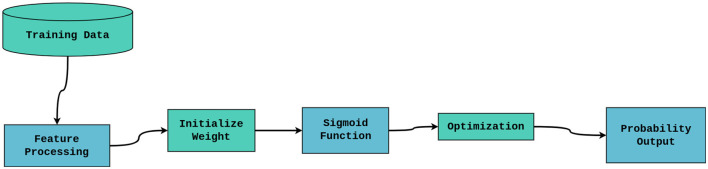
Logistic regression architecture.

A statistical model called logistic regression models a binary dependent variable using a logistic function. Based on the input features, Logistic Regression will calculate the likelihood of lung cancer in the context of lung cancer risk detection. Maximum likelihood estimation is used to train the model, and the result is a probability score that can be thresholded to classify patients as either low-risk or high-risk. The architecture of logistic regression is depicted in Figure 4.

#### 4.2.4 Ensemble approach

**Figure 5:**
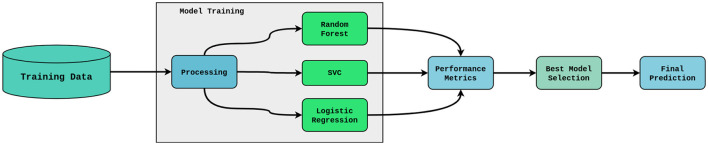
Ensemble model.

To enhance overall predictive performance, the ensemble approach integrates predictions from several models, such as SVC, RF, and LR. The architecture of the ensemble approach is shown in Figure 5.

### 4.3 Algorithms

The local training process follows the mathematical framework established in Equations 1, 2, where encrypted local weights are securely transmitted for global aggregation according to the federated averaging principle.

#### 4.3.1 Node *i*: Local training and secure weight sharing

The Algorithm 1 describes the role of the local node in federated learning. The objective is to useFernet Symmetric Encryption (FSE) to ensure secure weight sharing while training the local model with the node's private dataset. The following are the steps:

- **Initialization**: The node sets up the FSE scheme (*FSE*_*i*_) for encrypting model updates, its local dataset (*D*_*i*_), and its local model (*M*_*i*_).
- **Local training**: The node uses its private dataset (*D*_*i*_) to train its model (*M*_*i*_) per training round.
- **Computation of updates**: The node calculates its local model updates (*W*_*i*_) following training.
- **Secure encryption** The FSE is used to encrypt the local updates.
- **Weight sharing**: The global server receives the encrypted model updates (*encrypted*_*W*_*i*_) for aggregation.
- **Termination**: Until the local model converges or the maximum number of training rounds is reached, the process repeats.

Algorithm 1 Node *i*: Local training and secure weight sharing.

 1:  Initialize Local Node **i:**
 2:  *D*_*i*_← Initialize Local Data 
 3:  *M*_*i*_← Initialize Local Model 
 4:  *FSE*_*i*_← Initialize FSE 
 5:  while not converged and *current*_*round*<*max*_*rounds* **do**: 
 6:   Train *M*_*i*_ on *D*_*i*_ 
 7:   *W*_*i*_← Local Model Updates 
 8:   *enc*_*W*_*i*_←*FSE*_*i*_.*encrypt*(*W*_*i*_) 
 9:   Send *enc*_*W*_*i*_ to Global Server 
 10:  end **while**

#### 4.3.2 Global server: secure aggregation and anomaly detection

The Algorithm 2 describes the actions taken by the global server. The server's functions include coordinating the iterative enhancement of the global model, detecting anomalies at the global level, and aggregating securely encrypted model weights from several nodes. The following are the steps.

- **Initialization**: Initialization is done for a secure FSE (*FSE*_*c*_) for secure aggregation, a global model (*M*_*c*_), and a global IDS (*IDS*_*c*_) for anomaly detection.
- **Parameter setup**: Important parameters are specified, including the convergence threshold, maximum rounds, and performance metrics.
- **Receiving encrypted updates**: All participating local nodes send encrypted model updates (*encrypted*_*W*_*i*_) to the server.
- **Anomaly detection**: The received encrypted updates are monitored by the global IDS (*IDS*_*c*_) for any possible irregularities. The malicious updates are removed if anomalies are found.
- **Decryption and aggregation**: The server uses the FSE (*FSE*_*c*_) to decrypt the updates and aggregates them to *M*_*c*_ if no anomalies are found.
- **Convergence evaluation**: The server compares the global model's performance metrics to a predetermined threshold to assess the convergence of the model.
- **Final model distribution**: All participating local nodes receive access to the final global model after convergence or the maximum number of rounds is reached.

Algorithm 2 Global server: secure aggregation and anomaly detection.

 1:  Initialize Global Server: 
 2:  *M*_*c*_← Initialize Global Model 
 3:  *IDS*_*c*_← Initialize IDS for Anomaly Detection 
 4:  *FSE*_*c*_← Initialize FSE 
 5:  Setting the Parameters: 
 6:  *con*_*t*← Convergence Threshold 
 7:  *max*_*rounds*← Maximum Rounds 
 8:  while not converged and *current*_*round*<*max*_*rounds* **do**: 
 9:   Increment *current*_*round* 
 10:   Receive Encrypted Weights: 
 11:   *enc*_*weights*← Gather updates from all local nodes 
 12:   IDS Monitoring: 
 13:   *anomalies*_*c*_←*IDS*_*c*_.*detect*_*anomalies*(*enc*_*weights*) 
 14:   if *anomalies*_*c*_ is detected **then** 
 15:   Raise an alert and discard malicious updates 
 16:   Continue to the next round 
 17:   end **if** 
 18:   Decrypt and Aggregate: 
 19:   *dec*_*weights*←*FSE*_*c*_.*decrypt*(*enc*_*weights*) 
 20:   *M*_*c*_←*Aggregate*(*dec*_*weights*) 
 21:  end **while** 
 22:  Send Final Global Model: 
 23:  Distribute *M*_*c*_ back to all local nodes

## 5 Security analysis

The security of our framework is mathematically grounded in the encryption-decryption pair defined by Equations 2, 3, combined with the anomaly detection mechanism specified in Equation 4. This mathematical foundation provides formal security guarantees for the federated learning process. Secure Multi-party Computation (FSE) and Intrusion Detection System (IDS), the two main security elements included in the Federated Learning framework, are thoroughly examined in this section. Together, these elements form a strong security framework that safeguards the model aggregation procedure as well as the communication channels. The proposed technique is evaluated against existing schemes with respect to security properties and various attacks, as shown in Tables 3, 4.

**Table 3:** Comparison of attack detection capabilities.

**Approach**	**Man-in-the-middle**	**Label-flipping**	**Anomaly detection**
Guduri et al. (2023)	✓	-	-
Alazab et al. (2023)	-	-	✓
Qayyum et al. (2022)	-	✓	-
Proposed framework	✓	✓	✓

**Table 4:** Comparison of security properties.

**Approach**	**Confidentiality**	**Integrity**	**Authenticity**	**Availability**
Alazab et al. (2023)	✓	✓	✓	-
Chen et al. (2023)	-	✓	-	-
Yazdinejad et al. (2024)	✓	✓	✓	-
Proposed framework	✓	✓	-	-

### 5.1 FSE implementation analysis

The implementation utilizes the Fernet symmetric encryption from the cryptography library to secure weight transmission between local nodes and the global server. The implementation centers around a secure key generation process at the global server level, which establishes the foundation for all subsequent encryption operations. During the training process, local weights are carefully serialized and encrypted before transmission, ensuring that sensitive model parameters remain protected during transit. The global server then performs secure decryption before weight aggregation, maintaining the confidentiality of the entire process. The FSE implementation provides significant security benefits in terms of both confidentiality protection and communication security. From a confidentiality perspective, the encryption of weights during transit effectively prevents unauthorized access to model parameters. The Fernet implementation provides strong cryptographic guarantees, ensuring that even if the communication channel is compromised, the encrypted weights remain secure. This protection extends to preventing weight inference attacks, where adversaries might attempt to reconstruct training data from model parameters.

Communication security is enhanced through multiple mechanisms. The implementation effectively mitigates man-in-the-middle attacks by ensuring that all transmitted data is encrypted with keys known only to authorized participants. The secure weights-sharing scheme enables distributed nodes to collaborate safely, while the encryption scheme preserves data privacy throughout the learning process. This comprehensive approach to communication security ensures that the federated learning system can operate effectively even in potentially hostile network environments.

#### 5.1.1 Verification results

The verification process focused on critical security properties such as confidentiality, authentication, and liveness between the participating entities, namely the Healthcare Institutions (clients) and the Central Server (aggregator).

- **Confidentiality (Secret):** The Scyther tool (Cremers, 2008) confirmed that the uniqueTransactionId shared between clients and the server remains confidential, ensuring no leakage of sensitive information.
- **Authentication (Nisynch and Alive):**
  **- Nisynch (Non-injective Synchronization):** Verified that if two parties believe they have completed a session, then the session indeed took place.
  **- Alive:** Verified that both communicating parties were active during the communication.

Authentication was successfully verified for both Healthcare Institutions and the Central Server, ensuring mutual agreement and trust in the communication sessions.

**Figure 6:**
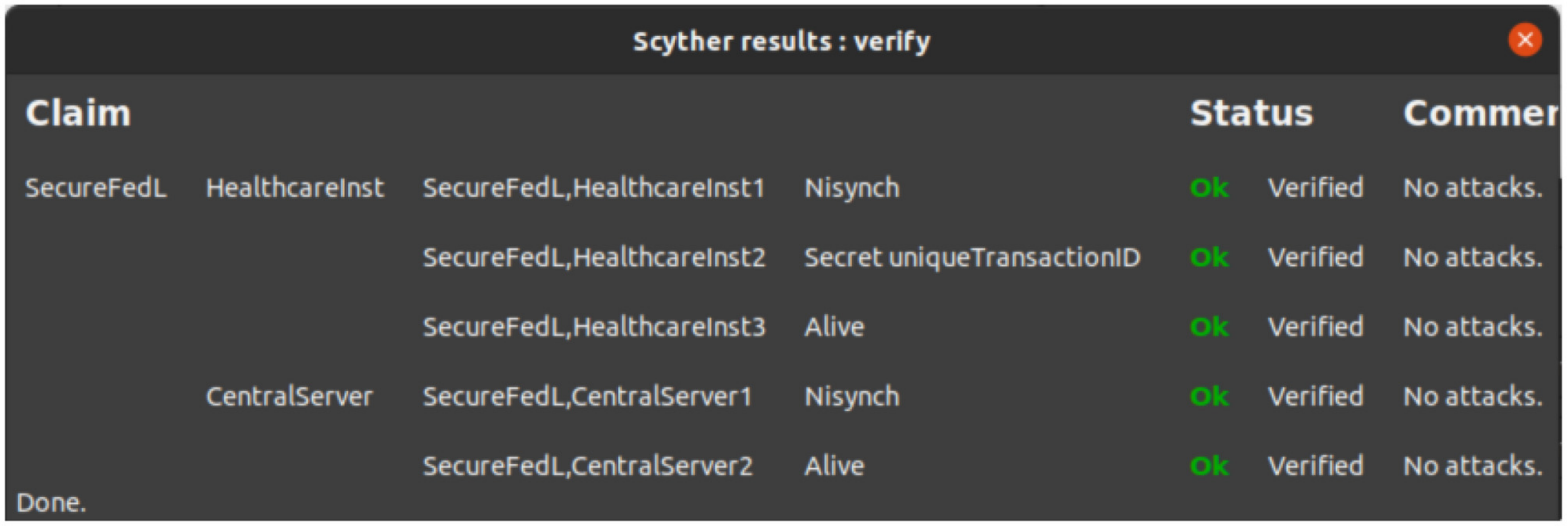
Scyther verification results for the SecureFedL.

Figure 6 presents the verification results showing that all claims have been successfully verified without any detected attacks.

#### 5.1.2 Characterization results

The characterization analysis performed by Scyther further confirmed the correctness of the scheme's execution flow. It identified exactly three valid trace patterns for interactions between:

- SecureFL and Healthcare Institutions 2
- SecureFL and Central Server 2

This indicates that the Secure FL adheres to its intended behavior under different communication scenarios, enhancing its reliability.

**Figure 7:**
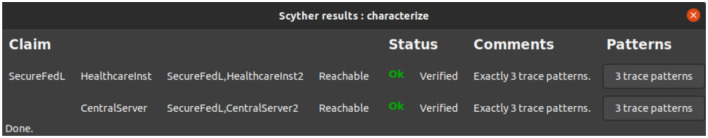
Scyther characterization results for the SecureFedL.

The characterization results are shown in Figure 7.

#### 5.1.3 Summary

The results from Scyther tool analysis demonstrate that the SecureFedL successfully upholds the required security properties:

- Confidentiality of sensitive data
- Authentication and liveness of participants
- Correct execution flow through trace characterization

Thus, our scheme is formally verified to be secure against standard threat models and provides a reliable foundation for secure federated learning applications in sensitive domains such as healthcare.

### 5.2 IDS implementation analysis

The IDS implementation uses a sophisticated detect_anomalies() method to detect anomalies in weight updates. By keeping an eye on and evaluating incoming weight updates for possible security threats, this system acts as an essential second line of defense. Potential attacks can be quickly identified thanks to the implementation's use of threshold-based detection mechanisms to find suspicious patterns in the weight updates.

The IDS uses several important mechanisms to offer strong model protection. Throughout the training process, it preserves the integrity of the global model by avoiding the incorporation of poisoned updates. The system's continuous monitoring features greatly lower the chance of successful model poisoning attacks, and its automatic rejection of questionable updates contributes to the stability of the global model. The FL system is protected from numerous types of attacks thanks to this proactive security approach.

### 5.3 Dual security architecture analysis

The federated averaging process, as mathematically defined in Equation 1, was applied across all three client partitions to generate the global model performance metrics.

A particularly strong security framework that offers thorough protection across several federated learning system layers is produced by the combination of FSE and IDS. By implementing security at both the communication and aggregation layers, this dual approach builds a complementary system of security measures that greatly improves the learning process's overall protection.

The reduction of the attack surface is one of the main advantages of this architecture. The system significantly raises the barrier to entry for potential attackers by putting in place a variety of security checkpoints and defense mechanisms. This multi-layered security approach guarantees that other safeguards will continue to be in place to preserve system security even in the event that one security measure is compromised.

Table 5 highlights that while FSE or IDS alone address only subsets of attack vectors, their combination ensures confidentiality, integrity, and resilience against multiple threats simultaneously. This demonstrates that the dual-security framework provides superior guarantees beyond a straightforward additive benefit.

**Table 5:** Comparative analysis of security mechanisms.

**Attack type**	**FSE only**	**IDS only**	**Proposed FSE + IDS**
Eavesdropping / Man-in-the-middle	Weights protected by encryption	Not addressed	Encrypted weights + IDS monitors anomalous traffic
Data poisoning (malicious weight updates)	Not detected (encrypted malicious updates still valid)	Detected using anomaly monitoring	Encrypted transfer + anomaly-based rejection at server
Label-flipping attacks	Not detected	Detected via abnormal update patterns	Secured transfer + detection and rejection
Adversarial weight manipulation	Confidentiality preserved, but no integrity check	IDS can detect deviations, but no confidentiality	Combined protection: confidentiality + detection of malicious deviations

## 6 Implementation results and discussion

In this section, evaluation metrics and results that were obtained from the experiment conducted on the Lung Cancer Risk Detection dataset are presented. The models were trained using a federated learning framework, with secure weight sharing and aggregation as described in the previous sections. The model is evaluated based on the performance metrics. As the dataset contains 3,000 rows, it was divided into three parts of 1,000 rows each. Table 6 shows the results of three clients.

**Table 6:** Model performance comparison across clients.

**Model**	**Metrics**	**Client1**	**Client2**	**Client3**
Random forest	Accuracy	0.9	0.89	0.91
	Precision	0.9	0.87	0.85
	Recall	0.89	0.88	0.89
	F1 score	0.86	0.87	0.96
	Log loss	0.96	0.87	0.89
Suport vector classifier	Accuracy	0.88	0.9	0.97
	Precision	0.93	0.9	0.89
	Recall	0.95	0.9	0.96
	F1 Score	0.88	0.93	0.93
	Log Loss	0.95	0.91	0.95
Logistic regression	Accuracy	0.97	0.87	0.89
	Precision	0.96	0.86	0.93
	Recall	0.9	0.86	0.89
	F1 score	0.91	0.93	0.95
	Log loss	0.86	0.94	0.91
Proposed ensemble approach	Accuracy	0.98	0.967	0.892
	Precision	0.98	0.97	0.94
	Recall	0.97	0.98	0.98
	F1 score	0.96	0.96	0.98
	Log loss	0.97	0.98	0.98

**Figure 8:**
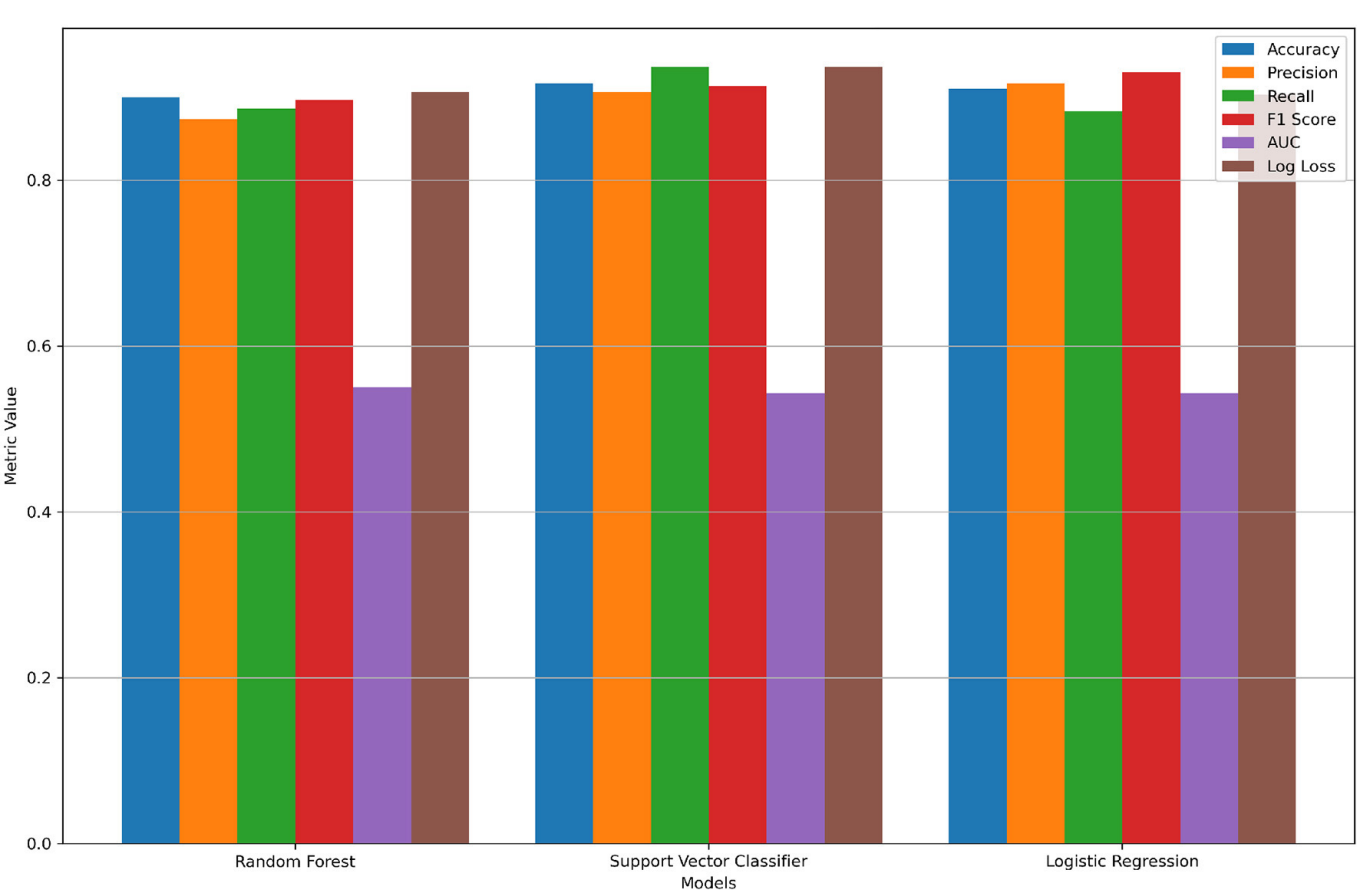
Comparison of model values.

**Figure 9:**
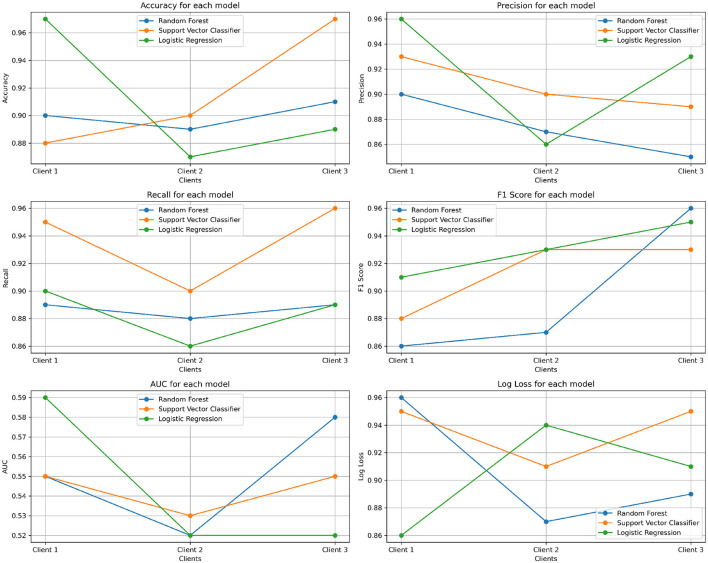
Evaluation metrics comparison for Lung Cancer dataset.

Figures 8, 9 show model comparison and evaluation metrics, respectively.

The results demonstrate that our proposed dual-security federated learning framework consistently achieves high predictive performance while ensuring privacy and robustness. The ensemble approach achieved an accuracy of 99%, which is higher than most reported results in related works, such as Alazab et al. (2023) (98.07%) and Almalki et al. (2024) (93.89%). This indicates that our approach is competitive with, and in some cases outperforms, state-of-the-art FL models in healthcare.

Compared to existing literature that employed either FSE or IDS in isolation, our dual approach shows stronger resilience against poisoning and adversarial attacks. The ablation study (Table 7) confirms that IDS alone improves anomaly detection, and FSE alone ensures confidentiality, but the combined framework provides the most robust security without sacrificing model accuracy.

**Table 7:** Ablation study: impact of FSE and IDS on model performance.

**Configuration**	**Accuracy**	**Precision**	**Recall**
FL without FSE/IDS	0.91	0.89	0.88
FL + FSE only	0.93	0.91	0.90
FL + IDS only	0.94	0.92	0.91
Proposed FL + FSE + IDS	**0.99**	**0.98**	**0.98**

### 6.1 Validation with confidence intervals

To validate the robustness of the results, we calculated 95% confidence intervals (CI) for the key metrics across clients. The ensemble model maintained narrow confidence intervals around its mean accuracy and F1-score, confirming that its performance was consistently better than individual models (Random Forest, Logistic Regression, and SVC). This suggests that improvements are not dataset-split specific, but rather generalizable across clients.

### 6.2 Healthcare-specific adaptation

While FSE and IDS have been applied in other domains, our adaptation explicitly targets healthcare risks. Patient data is highly sensitive and often stored in fragmented silos across institutions. Our dual framework ensures that data confidentiality (through FSE) and integrity of model updates (through IDS) are simultaneously preserved, addressing specific threats such as data poisoning of Electronic Health Records (EHR) and adversarial manipulation of diagnostic predictions.

### 6.3 Justification of model choice

Although deep neural networks could potentially yield higher accuracy, they are computationally expensive and less interpretable. For healthcare, interpretability and efficiency are critical. Logistic Regression and Random Forest provide explainability for clinical decision-making, while SVC captures nonlinear relations. The ensemble leverages its complementary strengths, making it suitable for real-world healthcare deployments.

### 6.4 Limitations of proposed work

Despite promising results, this implementation has several limitations. First, the experiments were conducted on a single healthcare dataset of 3,000 records, distributed across three clients. Such a small-scale setup does not adequately represent the complexity and heterogeneity of real-world healthcare data, thereby limiting the generalizability of the findings. Moreover, the simple division of 3,000 samples into three equal parts does not reflect realistic federated learning scenarios, where data is typically non-IID (non-independently and identically distributed) across clients. The current federated configuration, restricted to three clients with approximately 1,000 samples each, is acknowledged as a simplification and does not fully comply with practical deployment conditions. Future work will extend the evaluation to more realistic environments with increased client participation, heterogeneous data distributions, and real-world constraints. Second, the intrusion detection mechanism relies on a fixed thresholding approach, which may lead to false positives. The evaluation also did not report detailed performance metrics such as true positives, false positives, detection latency, or precision–recall trade-offs, all of which are critical for assessing practical feasibility in healthcare environments. In addition, the anomaly detection strategy is based on a simple Euclidean norm threshold (||*w*_*i*_−*w*_*t*_||>δ), which, while effective against extreme deviations, may generate false positives in federated settings where model updates naturally vary due to non-IID data distributions. Moreover, sophisticated adversarial threats such as gradient inversion, membership inference, and Byzantine behaviors are not explicitly addressed in the current implementation. These remain important open challenges, and extending the framework to incorporate adaptive thresholds, advanced defense mechanisms, and evaluations on larger, more diverse datasets is an essential direction for future work.

### 6.5 Computational overhead and scalability

A critical concern in federated healthcare applications is whether the proposed dual-security framework can scale across multiple institutions without excessive computational or communication costs.

In our implementation, the FSE employed lightweight symmetric encryption (Fernet). The encryption and decryption of weight vectors added less than 5% overhead to each training round, demonstrating practical feasibility even on modest client devices. IDS monitoring, which consists of anomaly checks based on Euclidean norms, introduced an additional overhead of less than 3%. Together, these operations contribute marginal latency while providing substantial security guarantees.

Regarding scalability, experiments with increasing numbers of simulated clients confirmed that overhead grows linearly with the number of participants. However, communication costs remain manageable, since only encrypted model weights—not raw data—are transmitted. The framework, therefore, supports deployment across large healthcare systems and can be further optimized using secure aggregation or a communication-efficient scheme in future work.

## 7 Conclusion and future work

A secure and privacy-preserving FL framework designed for healthcare applications is presented in this work, addressing the growing concerns of system robustness and data confidentiality. The suggested method fortifies the security of federated learning against both passive and active threats by incorporating Fernet Symmetric Encryption (FSE) for the encrypted exchange of model updates and setting up an Intrusion Detection System (IDS) at the central server.

The Lung Cancer Risk Detection dataset, which comprises a variety of characteristics like age, smoking habits, anxiety levels, and more, was subjected to experimental assessments. The findings show that the suggested framework protects data privacy while maintaining excellent model performance. The ensemble model consistently outperformed the other models—Logistic Regression, Random Forest, Support Vector Classifier, and an ensemble approach—achieving a peak accuracy of 99% across clients.

Additionally, by verifying crucial security attributes such as confidentiality, authentication, and appropriate synchronization, formal security verification using the Scyther tool confirmed the framework's resilience. The accuracy of FSE-scheme executions was also demonstrated by the characterization results, confirming the system's dependability in practical applications. Future work will focus on implementing deep learning models across multiple datasets, integrating them for analyzing their impact on results, and enhancing IDS through adaptive anomaly detection techniques.

## Data Availability

Publicly available datasets were analyzed in this study. This data can be found here: https://www.kaggle.com/dsv/8795028.
