# Supplementary material for: EnDuSecFed: an ensemble approach for privacy preserving Federated Learning with dual-security framework for sustainable healthcare
Source: Front Big Data. 2026 Jan 22;8:1659026. doi: 10.3389/fdata.2025.1659026 (PMC12878652; doi:10.3389/fdata.2025.1659026)
Supplement: Supplementary file 1 [file Data_Sheet_1.pdf]

Supplementary Material for

EnDuSecFed: An Ensemble approach for Privacy Preserving Federated Learning with Dual-security Framework for sustainable Healthcare

Bela Shrimali [1,*], Jenil Gajjar [2], Swapnoneel Roy [3,*], Sanjay Patel [4], Kanu Patel [5],
and Ramesh Ram Naik [6]

A Mathematical Symbols

This section provides a comprehensive definition of all mathematical symbols used throughout this article.

A.1 Mathematical Operations and Functions

- **Federated Averaging:** The global model weights are computed using the equation:

$$W_{\text{global}} = \frac{1}{N} \sum_{i=1}^{N} W_{\text{local},i}$$

  where the summation aggregates all local weights and division by N provides the average.

- **Encryption Operation:** Secure weight encryption is performed as:

$$W_{\text{encrypted}} = \text{Encrypt}(W_{\text{local}}, K_{\text{FSE}})$$

- **Decryption Operation:** Weight decryption at the server:

$$W_{\text{decrypted}} = \text{Decrypt}(W_{\text{encrypted}}, K_{\text{FSE}})$$

- **Anomaly Detection Condition:**

$$\text{Anomaly Detected} \quad \text{if} \quad \|\mathbf{w}_i - \mathbf{w}_t\| > \delta$$

  This condition triggers when the Euclidean distance between client weights and global weights exceeds the threshold.

Table 1: Mathematical Symbols and Definitions

Symbol	Definition
W_{global}	Global model weights after aggregation
W_{local}	Local model weights at client node
$W_{\text{local},i}$	Local model weights of client i
W_i	Local model weights/updates of node i
$W_{\text{encrypted}}$	Encrypted model weights
$W_{\text{decrypted}}$	Decrypted model weights
enc_W_i	Encrypted weights of node i
$encrypted\_W_i$	Encrypted model updates from node i
N	Total number of participating clients/nodes
K_{FSE}	Encryption key used in FSE
$\mathbf{w}_i$	Weight vector of client i
$\mathbf{w}_t$	Global model weight vector at time t
δ	Predefined threshold for anomaly detection
$\|\cdot\|$	Euclidean norm operator
D_i	Private dataset of node i
M_i	Local model of node i
M_c	Global model at central server
FSE_i	FSE instance of node i
FSE_c	FSE instance of central server
IDS_c	Intrusion Detection System instance at central server
con_t	Convergence threshold parameter
max_rounds	Maximum number of training rounds
$current\_round$	Current training round number
$enc\_weights$	Collection of encrypted weights from all nodes
$dec\_weights$	Collection of decrypted weights at server
$anomalies_c$	Detected anomalies at central server
i	Index representing client/node identifier
$\text{Encrypt}(\cdot,\cdot)$	Encryption function with data and key parameters
$\text{Decrypt}(\cdot,\cdot)$	Decryption function with encrypted data and key
$\sum_{i=1}^{N}$	Summation operator over all N clients
$\frac{1}{N}$	Averaging factor for federated aggregation

A.2 Notation Conventions

- Subscript i denotes client/node-specific parameters
- Subscript c denotes central server parameters
- Subscript global denotes aggregated global parameters
- Subscript local denotes node-local parameters
- Bold symbols ($\mathbf{w}$) represent vector quantities
- Regular symbols (W) represent scalar or matrix quantities

Table 2: Algorithm Notation and Control Structures

Notation	Definition
$\leftarrow$	Assignment operator (assigns value to variable)
$<$	Less than comparison operator
$>$	Greater than comparison operator
not converged	Boolean condition for model convergence status
and	Logical AND operator
if	Conditional statement
while	Iterative loop construct
Send	Data transmission operation
Gather	Data collection operation
Aggregate($\cdot$)	Model weight aggregation function
Distribute	Model distribution operation
Increment	Counter increment operation
Continue	Loop continuation command
Raise an alert	Security alert generation
Discard	Data rejection operation

Table 3: Security and Detection Symbols

Symbol	Definition
Anomaly Detected	Boolean indicator for anomaly presence
is detected	Detection confirmation operator
$FSE_i.encrypt(\cdot)$	FSE encryption method for node i
$FSE_c.decrypt(\cdot)$	FSE decryption method at central server
$IDS_c.detect\_anomalies(\cdot)$	Anomaly detection method at server
uniqueTransactionId	Unique identifier for secure transactions
	Symbol indicating feature presence/support
–	Symbol indicating feature absence/not supported

- Underscored symbols (enc_W_i) represent processed/transformed data
